# Tissue-infiltrating immune cells as prognostic markers in oral squamous cell carcinoma: a systematic review and meta-analysis

**DOI:** 10.1038/s41416-019-0409-6

**Published:** 2019-02-27

**Authors:** Elin Hadler-Olsen, Anna Maria Wirsing

**Affiliations:** 1 Department of Medical Biology, Faculty of Health Sciences, University of Tromsø – The Arctic University of Norway, 9037 Tromsø, Norway; 2 Department of Clinical Dentistry, Faculty of Health Sciences, University of Tromsø – The Arctic University of Norway, 9037 Tromsø, Norway; 3 Department of Clinical Pathology, University Hospital of North Norway, 9038 Tromsø, Norway

## Abstract

**Background:**

Various immune cells have been suggested as prognostic markers for cancer patients. In this article, we present a systematic review and meta-analysis of studies assessing the prognostic value of tissue-infiltrating immune cells in oral cancer and discuss the reporting quality of these studies.

**Methods:**

We performed a systematic literature search and included studies using immunohistochemistry and survival analysis to assess the prognostic value of tumour-infiltrating T cells, B cells, macrophages, dendritic cells, mast cells and natural killer cells in oral cancer. We performed meta-analysis of studies providing necessary statistical data and investigated the studies’ adherence to the REporting recommendations for tumour MARKer prognostic studies (REMARK) guidelines.

**Results:**

Of the 1960 articles identified, 33 were eligible for this systematic review and 8 were included in the meta-analysis. CD163+ M2 macrophages and CD57+ natural killer cells were the most promising predictors of survival in oral cancer patients. Many studies lacked important information on their design and conduct.

**Conclusion:**

Deficiencies in the reporting of study design and conduct make it difficult to draw reliable conclusions about the suggested markers. The prognostic value of CD163+ M2 macrophages and CD57+ natural killer cells should be validated in large, standardised studies.

## Background

Squamous cell carcinomas (SCC) account for the vast majority of oral (O) cancer.^1^ Surgery, often accompanied by radiotherapy, is the standard treatment for these tumours.^2^ The radiation causes severe, chronic side effects including xerostomia and problems with speech, oral intake and dental health, which makes it important to avoid overtreatment.^3^ Currently, the most reliable prognostic factor for OSCC patients is the TNM classification system, which stages cancers according to the tumour size and depth of invasion (T), the presence and extent of regional lymph node metastases (N), and the presence of distant metastases (M).^4^ The individual TNM categories can be grouped into stages I–IV reflecting improved survival for patients with early- compared to advanced-stage tumours.^4^ Nevertheless, tumours of the same stage are heterogeneous with respect to aggressiveness and response to therapy. Thus, the TNM classification needs reinforcement with biomarkers that more reliably reflect the biological diversity of these tumours to better tailor the treatment to the patient’s need.

The promising results of immuno-modulating therapies such as PD-1/PD-L1-blocking antibodies demonstrate that the immune system is significantly involved in tumour progression,^5,6^ and have boosted the interest in tumour immunology. However, the immune system is complex, and infiltrating immune cells may exert various roles in different types of cancer as well as within different sub-locations of a single tumour.^7–9^ Although a plethora of immune-biomarkers have been launched as useful prognosticators for OSCC patients,^10–12^ none of them have been generally accepted and implemented in clinical practice. Major concerns have been raised about the poor quality of many biomarker studies.^10,13^ The US National Cancer Institute and the European Organization for Research and Treatment of Cancer initiated the development of the REporting recommendations for tumour MARKer prognostic studies (REMARK) guidelines, which were in 2005 simultaneously published in five cancer-related international journals.^14^ These guidelines are a 20-item checklist outlining the minimum information and analyses needed in prognostic marker studies to ensure quality, reproducibility and opportunity to pool studies in meta-analyses.

Recognising the involvement of the immune system in cancer and the need for reliable prognostic markers for OSCC, we have conducted a systematic review and meta-analysis of studies assessing the prognostic value of tissue-infiltrating immune cells in OSCC by survival analysis. We have included studies employing immunohistochemistry to detect one or several of the following immune cells: T cells, B cells, macrophages, dendritic cells (DC), mast cells and natural killer (NK) cells. We have also assessed to what extent the clinical and pathological data, immunohistochemical staining and scoring procedures as well as results were adequately described in the reviewed papers, according to the REMARK guidelines.^14^ Proper reporting of these parameters allows the reader to evaluate the quality and reliability of the results, and may help to guide cancer biomarker research in the right direction.

## Methods

### Eligibility criteria

Included in the review were original articles that fulfilled all the following criteria, as further elaborated in the text below:

- were written in English,
- presented data from patients with SCC in the oral cavity proper,
- analysed tissue that had not been previously exposed to radiotherapy and/or chemotherapy,
- used immunohistochemistry on tumour tissue sections to recognise the immune cells of interest,
- addressed the prognostic value of tumour-associated macrophages, DC, NK cells, mast cells, T cells and/or B cells by univariate and/or multivariate survival analyses of at least 40 OSCC patients, and
- employed some kind of survival as endpoint in the survival analyses

Cancers from different sites of the head and neck region, such as the oral cavity, the oropharynx and larynx have distinct subsite characteristics,^15^ and should therefore be treated as different entities. Thus, we only included studies that reported specific survival data for at least 40 patients with SCC in the oral cavity proper. Notably, the cut-off for the number of patients is based on what we think is a reasonable cohort size to include in biomarker studies and does not derive from the statistical analysis. Survival endpoints with unclear or missing information were included and interpreted as overall survival.

### Sources of information, search terms and screening

**Fig. 1:**
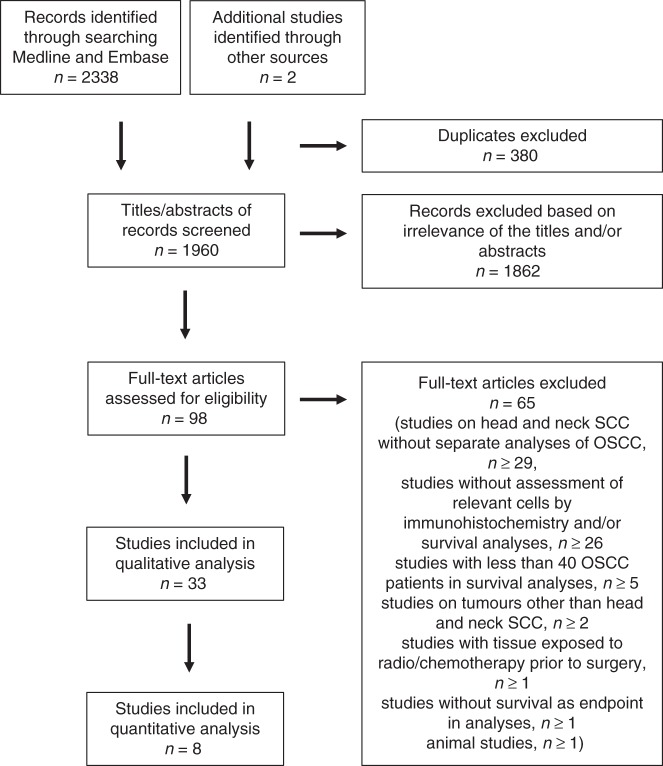
Flow chart demonstrating reasons for exclusion of studies identified in the searches

We searched Embase and Medline on the 14th of March 2018 using the Ovid interface with the search terms given in Supplementary Figure 1. If applicable, our entry terms were defined based on Medical Subject Headings (MeSH) from Pubmed^16^ to develop a controlled vocabulary. In addition to MeSH terms, we included relevant free-text entry terms. We defined three sets of entry terms describing (1) prognostic value, (2) selected immune cells and (3) oral cancer. The search terms within each set were combined with the Boolean operator OR, and the three sets were then combined with the Boolean operator AND. In addition, we searched the Cochrane Library and the reference lists of reviews on related topics^5,17–21^ to look for additional relevant papers. We automatically discarded duplicate articles, non-English literature and non-human studies in Medline and Embase. The two review authors independently extracted relevant articles based on title and abstract. The full-text papers were screened for all papers that appeared to meet the inclusion criteria or in case of uncertainty. In case of uncertainty or discrepancy, we reached consensus by analysis together. We designed a flow diagram with the excluded cases (Fig. 1) adherent to the Preferred Reporting Items for Systematic Review and Meta-Analysis (PRISMA) guidelines.^22^

### Data collection

For all studies included, we retrieved the following information when available: name of the first author, year of publication, number of patients, the tumour sizes or stages included, the country and period in which the patient cohort was gathered, the tumour compartment analysed, the primary antibody used and survival data. If the papers reported survival data over time, e.g. by Kaplan–Meier curves, we recorded whether tumour infiltration of the various immune cells was associated with longer or shorter survival, independent of the statistical significance of the presented results. We retrieved the following statistical data when available: statistical results from univariate and multivariate analysis including the estimated hazard ratio (HR) or risk ratio (RR), the associated 95% confidence interval (CI) and the *P*-value.

### Meta-analysis

**Fig. 2:**
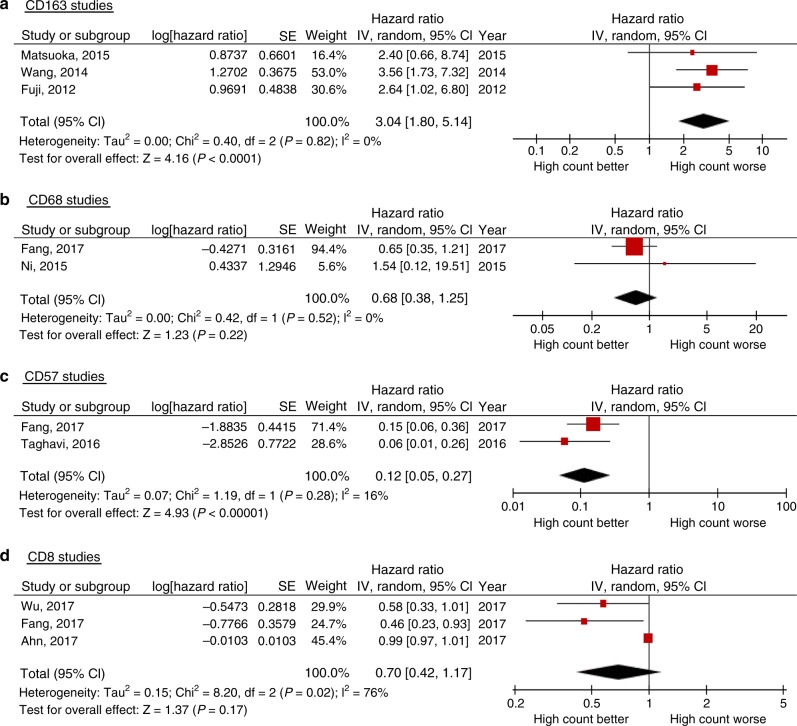
Forest plots illustrating the results of meta-analysis of studies assessing the prognostic value of **a** CD163-positive, **b** CD68-positive, **c** CD57-positive and **d** CD8-positive tissue-infiltrating immune cells in oral squamous cell carcinoma

We used a random-effect meta-analysis of overall survival to estimate the summary HR and the associated 95% CI for immune cell markers that had been reported in at least two studies with necessary statistical data (either univariate or multivariate estimates of HR, and the associated 95% CI). The meta-analysis was based on multivariate estimates of HR, as this was the most commonly reported variable, except in the study by Ahn et al.^23^ where only the univariate estimate of HR was available. The direction of HR was high vs low for all immune cells analysed. If the HR estimate was reported in the opposite direction, we inverted the HR and CI. We quantified the heterogeneity of HRs across studies using *I*² statistics.^24^ Robustness of the statistical outcome of at least three studies was evaluated by assessing the effect of deleting each study in turn.^25^ The data presented in Fig. 2 were pooled using the generic inverse variance approach and the random effects model. Analyses were performed and forest plots created using Review Manager (RevMan) V.5.3.^26^

### Assessment of reporting quality

We assessed the reporting quality of the studies eligible for meta-analysis according to the REMARK guidelines.^14^ Of the original 20-item checklist, we chose three broad categories that we deemed pertinent for our review. Each study was judged based on the following broad categories: the study cohort, the immunohistochemical staining and scoring as well as the analysis and presentation of the results. The parameters evaluated for each of these categories were as follows: Study cohort: number of patients, tumour stage/size and chemotherapy /radiation exposure of tissue prior to surgery; Immunohistochemical staining: antibody clone/product number, immunohistochemistry procedures, positive and negative controls; scoring: number of observers, clear scoring criteria, inter/intra-observer variability; Analysis and presentation of the results: analysed survival endpoint, direction of effect on survival in Kaplan–Meier plot, estimated effects with CI for the marker, and at least for the final model, all other variables in the model.

## Results

We identified 1960 records in our search, of which 33 articles were eligible for this systematic review (Fig. 1). We conducted meta-analysis on overall survival for CD68, CD163, CD57 and CD8, as at least two studies provided the necessary statistical data (HR and associated 95% CI) for these biomarkers. The pooled estimates were calculated from two studies each for CD68 and CD57, and from three studies each for CD163 and CD8. The results of the systematic review are summarised in Tables 1–3, and the results of the meta-analysis are summarised in Fig. 2.

**Table 1:** Studies assessing the prognostic value of macrophages in OSCC

Authors, ref.	Number of patients, tumour size/stage, inclusion period, country	Tumour compartment analysed	Direction of effect on survival high cell count	Statistical significance	
				UV	MV
Marker: CD68, pan-macrophage marker					
Sun et al.^27^	72^a^, Stage I–IV, 2009-2011, China	No info	Negative	**OS:** ***P*** = 0.034	**OS:** ***P*** = 0.015
Wirsing et al.^28^	75, T1–T4, 1986–2002, Norway	Stroma at tumour front	Positive	**DSS:** ***P*** = 0.027	DSS: *P* > 0.05
Fang et al.^29^	78^a^, Stage I–IV, 2007–2009, China	Stroma	No info	OS: *P* = 0.293, HR 1.364 (95% CI 0.765–2.433)^b^	OS: *P* = 0.177, HR 0.652 (95% CI 0.351–1.212)^b^
Hu et al.^30^	127, Stage I–IV, 2007–2013, China	Nest	Negative	**OS:** ***P*** = 0.01, RR 3.08 (95% CI 2.236–3.914)	NA
		Stroma	Positive	OS: *P* = 0.3, RR .69 (95% CI −0.010–1.390)	NA
Sakakura et al.^31^	74, Stage I–IV, 2000–2012, Japan	Stroma	Negative	**OS:** ***P*** = 0.035	OS: *P* > 0.05
Ni et al.^32^	91, T1–T4, 2003–2011, China	Stroma	Negative	**OS:** ***P*** = 0.033, HR 1.947 (95% CI 1.512–10.379)	OS: *P* = 0.736, HR 1.55 (95% CI 0.122–19.515)
				DFS: *P* = 0.435	NA
		Nest	Positive	OS: *P* = 0.802, HR 0.904 (95% CI 0.180–4.552)	NA
				DFS: *P* = 0.562	NA
Costa et al.^33^	45, T1–T4, Period missing, Brazil	Peritumour	Negative	OS: *P* = 0.08	NA
Dayan et al.^34^	54, Stage I–IV, 1990–2006, Israel	Tumour–stroma interface	No info	OS: *P* > 0.05	NA
Fujii et al.^35^	108, Stage I–IV, 1990–2005, Japan	Stroma at invasive front	No info.	OS: *P* = 0.16	NA
Lu et al.^36^	92, Stage I–IV, 1995–2003, Taiwan	Stroma	Negative	**OS**: ***P*** < 0.001	**OS:** ***P*** = 0.015
				**DFS:** ***P*** = 0.001	**DFS:** ***P*** = 0.005
Liu et al.^37^	112^a^, T1–T4, Period missing, Taiwan	No info	Negative	**DFS:** ***P*** = 0.001	NA
Marker: CD163, M2 macrophages					
Kubota et al.^38^	46, Stage I–IV, 2005–2015, Japan	Nest	Negative	PFS: *P* = 0.21, HR 1.53 (95% CI 0.77–3.02)	PFS: *P* = 0.64, HR 1.18 (95% CI 0.57–2.42)
				DSS: *P* = 0.58, HR 1.22 (95% CI 0.57–2.50)	DSS: *P* = 0.61, HR 1.20 (95% CI 0.56–2.46)
Hu et al.^30^	127, Stage I–IV, 2007–2013, China	Nest	Negative	**OS:** ***P*** = 0.02, RR 2.83 (95% CI 1.991–3.669)	NA
		Stroma	Positive	OS: *P* = 0.48, RR 0.78 (95% CI 0.085–1.473)	NA
Sakakura et al.^31^	74, Stage I–IV, 2000–2012, Japan	Stroma	Negative	**OS:** ***P*** = 0.025	**OS:** ***P*** = 0.034
				**PFS:** ***P*** = 0.011	**PFS:** ***P*** = 0.023
Matsuoka et al.^39^	60, Stage I–IV, 2003–2009, Japan	Stroma at invasive front	Negative	**OS:** ***P*** = 0.003	OS: *P* = 0.195, HR 2.299 (95% CI 0.657–8.737)
				**DFS:** ***P*** = 0.007	DFS: *P* = 0.258, HR 1.749 (95% CI 0.669–4.904)
Fujita et al.^40^	50^a^, Stage I–IV, 2006–2010, Japan	Invasive front	Negative	**OS:** ***P*** = 0.006	OS: NA
				**DFS:** ***P*** = 0.002	**DFS:** ***P*** = 0.006, HR 2.625 (95% CI 1.312–5.253)
		Intratumour	No info.	OS: *P* > 0.05	NA
				DFS: *P* > 0.05	NA
Wang et al.^41^	240, Stage I–IV, M0, 1982–2005, China	Stroma	Negative	**OS:** ***P*** < 0.001, HR 4.411 (95% CI 2.578–7.547^a^)	**OS:** ***P*** = 0.001, HR 3.561 (95% CI 1.733–7.320)
Dayan et al.^34^	54, Stage I–IV, 1990–2006, Israel	Tumour–stroma interface	No info	OS: *P* > 0.05	NA
Fujii et al.^35^	108, Stage I–IV, 1990–2005, Japan	Stroma at invasive front	Negative	**OS:** ***P*** = 0.007	**OS**: ***P*** = 0.045, HR 2.636 (95% CI 1.021–6.803)

*OS* overall survival, if survival was not specified it was interpreted as overall survival, *DSS* disease-specific survival, *PFS* progression-free survival, *RFS* recurrence-free survival, *DFS* disease-free survival, *HR* hazards ratio, *RR* risk ratio, *NA* Not applied

Significant values are bold (*p*< 0.05)

^a^Denotes that information about chemotherapy/radiation exposure of tissue prior to surgery was missing or ambiguous

^b^Denotes that the HR and CI were inverted in these studies

**Table 2:** Studies assessing the prognostic value of dendritic cells, mast cells and natural killer cells in OSCC

Authors, ref.	Number of patients, tumour size/stage, Inclusion period, country	Tumour compartment analysed	Direction of effect on survival high cell count	Statistical significance	
				UV	MV
Dendritic cells					
Marker: CD1a, monocyte-derived DC and Langerhans cells					
Jardim et al.^56^	53, Stage I–IV, 2002–2010, Brazil	Intratumour	Positive	OS: *P* = 0.148	NA
				DFS: *P* = 0.089	NA
		Peritumour	Positive	**OS:** ***P*** = 0.03	**OS:** ***P*** = 0.001, HR 0.277 (95% CI 0.126–0.613)^b^
				**DFS:** ***P*** = 0.007	**DFS:** ***P*** = 0.001, HR 0.236 (95% CI 0.109–0.510)^b^
Sakakura et al.^45^	74, Stage I–IV, 2000–2012, Japan	Tumour periphery	No info	OS: *P* = 1.000	NA
				PFS: *P* = 1.000	NA
Goldman et al.^55^	43, T1–T4, 1987–1998, US	Peritumour	Positive	DSS: *P* = 0.05	DSS: *P* = 0.23
		Intratumour	Negative	DSS: *P* = 0.21	**DSS:** ***P*** = 0.04
Marker: S100, pan DC marker					
Reichert et al.^54^	132, Stage I–IV, 1980–1993, Germany	Stroma	Positive	**OS**: ***P*** < 0.001	**OS:** ***P*** ≤ 0.001, HR 0.422 (CI no info)
Goldman et al.^55^	43, T1–T4, 1987–1998, US	Peritumour	Positive	DSS: *P* = 0.30	DSS: *P* = 0.07
		Intratumour	Negative	DSS: *P* = 0.24	DSS: *P* = 0.80
Marker: CD83, mature DC					
Jardim et al.^56^	53, Stage I–IV, 2002–2010, Brazil	Intratumour	Positive	OS: *P* = 0.274	NA
				DFS: *P* = 0.346	NA
		Peritumour	Positive	OS: *P* = 0.276	NA
				DFS: *P* = 0.392	NA
Marker: P55, fascin-expressing DC					
Reichert et al.^54^	129, Stage I–IV, 1980–1993, Germany	Stroma	Positive	**OS:** ***P*** < 0.001	OS: *P* > 0.05
Marker: CD208/DClamp, mature DC					
Wirsing et al.^28^	69, T1–T4, 1986–2002, Norway	Stroma	Positive	DSS: *P* = 0.639	NA
Ni et al.^53^	79, Stage I–IV, 2011–2012, China	Nest	Positive	OS: *P* > 0.05	NA
		Stroma	Negative	OS: *P* > 0.05	NA
Marker: CD123, plasmacytoid DC, pre-DC					
O’Donnel et al.^57^	63^a^, T1–T4, Period missing, US	Extranestal	Negative/no info	**OS:** ***P*** < 0.0001,	NA
		Intratumour	Negative/no info.	Compartment unclear	NA
Marker: CD209/DCsign, immature DC					
O’Donnel et al.^57^	63^a^, T1–T4, Period missing, US	Extranestal	Negative/ no info	**OS:** ***P*** < 0.0001,	NA
		Intratumour	Negative/no info.	Compartment unclear	NA
Mast cells					
Marker: mast cell tryptase					
Akbarzadeh Baghban et al.^43^	57^a^, Stage missing, Period missing, Iran	Peritumour	No info	NA	OS: *P* = 0.719, HR 1.117 (95% CI 0.612–2.039)
Ishikawa et al.^59^	81^a^, Stage I–IV, 1982–2007, Japan	Stroma	Negative	**DFS:** ***P*** = 0.038	NA
Natural killer cells					
Marker: CD56, pan NK cell marker					
Sakakura et al.^45^	74, Stage I–IV, 2000–2012, Japan	Tumour periphery	No info	OS: *P* = 1.000 PFS: *P* = 1.000	NA
Marker: CD57, mature/activated NK cell marker					
Fang et al.^29^	78^a^, Stage I–IV, 2007–2009, China	Stroma	Positive	**OS:** ***P*** < 0.001, HR 0.130 (95% CI 0.061–0.274)^b^	**OS:** ***P*** < 0.001, HR 0.152 (95% CI 0.064–0.361)^b^
Taghavi et al.^43^	57^a^, Stage missing, Period missing, Iran	Intratumour	Positive	NA	**OS:** ***P*** < 0.001, HR 0.058 (95% CI 0.013–0.262)^b^
Zancope et al.^44^	40, Stage I–IV, Period missing, Brazil	Peritumour	No info	OS: *P* = 0.70	NA
		Intratumour	No info	OS: *P* = 0.69	

*OS* overall survival, if survival was not specified it was interpreted as overall survival, *DSS* disease-specific survival, *PFS* progression-free survival, *DFS* disease-free survival, *HR* hazards ratio, *NA* Not applied

Significant values are bold (*p*< 0.05)

^a^Denotes that information about chemotherapy/radiation exposure of tissue prior to surgery was missing or ambiguous

^b^Denotes that the HR and CI were inverted in these studies

**Table 3:** Studies assessing the prognostic value of T cells and B cells in OSCC

Authors, ref.	Number of patients, tumour size/stage, Inclusion period, country	Tumour compartment analysed	Direction of effect on survival high cell count	Statistical significance	
				UV	MV
T cells					
Marker: CD3, pan T cell marker					
Wirsing et al.^28^	74, T1–T4, 1986–2002, Norway	Stroma at tumour front	Positive	DSS: *P* = 0.200	NA
Ahn et al.^23^	68^a^, Stage I–IV, 2003–2011, South Korea	Stroma	Positive	OS: *P* = 0.142, HR 0.99 (95% CI 0.98–1.00)	NA
			No effect	DFS: *P* = 0.552, HR 1.00 (95% CI 0.98–1.01)	NA
Sakakura et al.^45^	74, Stage I–IV, 2000–2012, Japan	Tumour periphery	No info	OS: *P* = 0.856, PFS: *P* = 0.981	NA
Dayan et al.^34^	54, Stage I–IV, 1990–2006, Israel	Tumour–stroma interface	No info	OS: *P* > 0.05	NA
Marker: CD4, various T cell subsets					
Wirsing et al.^28^	72, T1–T4, 1986–2002, Norway	Stroma at tumour front	Positive	DSS: *P* = 0.691	NA
Fang et al.^29^	78^a^, Stage I–IV, 2007–2009, China	Stroma	No info	OS: *P* = 0.207, HR 1.458 (95% CI 0.812–2.618)^b^	OS: *P* = 0.909, HR 0.963 (95% CI 0.506–12.835)^b^
Mattox et al.^47^	47^a^, T1/T2, N0–N2, Period missing, US	No info	Positive	OS: *P* = 0.18	NA
Dayan et al.^34^	54, Stage I–IV, 1990–2006, Israel	Tumour–stroma interface	No info	OS: *P* > 0.05	NA
Cho et al.^46^	45, Stage I–IV, Period missing, South Korea	Peritumour	Positive	OS: *P* = 0.571	NA
Watanabe et al.^48^	87, Stage I–IV (M0), 1994–2003, Japan	Stroma	No info	OS: *P* = 0.072	NA
		Nest	No info	No info	NA
Marker: T-bet, Th1 cell marker					
Fang et al.^29^	78^a^, Stage I–IV, 2007–2009, China	Stroma	No info	OS: *P* = 0.639 h 1.148 (95% CI 0.645–2.045)^b^	OS: *P* = 0.836, HR 0.938 (95% CI 0.510–1.724)^b^
Marker: FoxP3+/− CCR4, Treg marker					
Ahn et al.^23^	68^a^, Stage I–IV, 2003–2011, South Korea	Stroma	Positive	OS: *P* = 0.374, HR 0.98 (95% CI 0.93–1.03)	NA
			Negative	DFS: *P* = 0.754, HR 1.01 (95% CI 0.96–1.06)	NA
Zhou et al.^49^	46, Stage I–IV, 2006–2011, China	Stroma	Negative	**OS:** ***P*** = 0.001	***OS: P*** **= **0.021, RR 15.152 (CI no info)^**b**^
Fujita et al.^40^	50^a^, Stage I–IV, 2006–2010, Japan	Invasive front	No info	OS: *P* > 0.05, DSS: *P* > 0.05	NA
		Intratumour	No info	OS: *P* > 0.05, DSS: *P* > 0.05	NA
Dayan et al.^34^	54, Stage I–IV, 1990–2006, Israel	Tumour–stroma interface	No info	OS: *P* > 0.05	NA
Watanabe et al.^48^	87, Stage I–IV (M0), 1994–2003, Japan	Stroma	Negative	OS: *P* = 0.31 (FoxP3)	No info
				**OS**: ***P*** = 0.001 (FoxP3/CCR4+)	No info
Marker: CD8, cytotoxic T cell marker					
Wirsing et al.^28^	72, T1–T4, 1986–2002, Norway	Stroma at tumour front	Negative	DSS: *P* = 0.304	NA
Ahn et al.^23^	68^a^, Stage I–IV, 2003–2011, South Korea	Stroma	Positive	OS: *P* = 0.181, HR 0.99 (95% CI 0.97–1.01)	NA
			Positive	DFS: *P* = 0.282, HR 0.99 (95% CI 0.96–1.01)	NA
Fang et al.^29^	78^a^, Stage I–IV, 2007–2009, China	Stroma	Positive	**OS:** ***P*** < 0.001, HR 0.263 (95% CI 0.138–0.501)^b^	**OS:** ***P*** = 0.030, HR 0.460 (95% CI 0.228–0.928)^b^
Kogashiwa et al.^50^	84, Stage III–IVA, 2007–2014, Japan	No info	Positive	OS: *P* = 0.058	NA
				PFS: *P* = 0.35	
Mattox et al.^47^	48^a^, T1/T2, N0–N2, Period missing, US	No info	No info	OS: *P* = 0.41	NA
Wu et al.^51^	165, T1–T4, 2008–2010 and 2012–2015, China	No info (TMA)	Positive	**OS:** ***P*** = 0.0498	OS: *P* = 0.052, HR 0.579 (95% CI 0.333–1.005)
Dayan et al.^34^	54, Stage I–IV, 1990–2006, Israel	Tumour–stroma interface	No info	OS: *P* > 0.05	NA
Cho et al.^46^	44, Stage I–IV, Period missing, South Korea	Intratumour	No info	No info	NA
		Peritumour	Positive	OS: *P* = 0.178	NA
Watanabe et al.^48^	87, Stage I–IV, M0, 1994–2003, Japan	Stroma	Positive	**OS:** ***P*** = 0.001	NA
		Nest	Positive	**OS**: ***P*** = 0.001	NA
Zancope et al.^44^	40, T1–T4, Period missing, Brazil	Peritumour	Positive	OS: *P* = 0.40	NA
		Intratumour	No info	OS: *P* = 0.9	NA
B cells					
Marker: CD20, pan B-cell marker					
Wirsing et al.^28^	75, T1–T4, 1986–2002, Norway	Stroma at invasive front	Positive	**DSS:** ***P*** = 0.002	DSS: *P* > 0.05
Ahn et al.^23^	68^a^, Stage I–IV, 2003–2011, South Korea	Stroma	Positive	OS: *P* = 0.186, HR 0.98 (95% CI 0.96–1.00)	NA
			Positive	DFS: *P* = 0.496, HR 0.99 (95% CI 0.97–1.02)	NA
Dayan et al.^34^	54, Stage II–V, 1990–2006, Israel	Tumour–stroma interface	No info	OS: *P* > 0.05	NA
Marker: CD19, pan B-cell marker					
Lao et al.^52^	93^a^, Stage I–IV, Period missing, China	Stroma	Positive	**OS:** ***P*** = 0.008	OS: Significant, but *P*-value and HR missing
Marker: CD138, plasmacell marker					
Dayan et al.^34^	64, Stage I–IV, 1990–2006, Israel	Tumour–stroma interface	No info	OS: *P* > 0.05	NA
Marker: IL19/CD19, Breg marker					
Zhou et al.^49^	46, Stage I–IV, 2006-2011, China	Stroma	Negative	**OS:** ***P*** = 0.001	OS: *P* = 0.528, RR 1.575 (CI no info)^b^

*OS* overall survival, if survival was not specified it was interpreted as overall survival, *DSS* disease-specific survival, *PFS* progression-free survival, *HR* hazards ratio, *RR* risk ratio, *NA* not applied

Significant values are bold (*p*< 0.05)

^a^Denotes that information about chemotherapy/radiation exposure of tissue prior to surgery was missing or ambiguous

^b^Denotes that the HR and CI were inverted in these studies

### CD163+ macrophages and CD57+ NK cells have prognostic potential

Macrophages were the most commonly studied cells of those addressed in this review^27–41^ (Table 1), and the majority of the studies reported a negative effect on survival of this cell type. A few studies scored macrophages infiltrating the tumour islands and the tumour–stroma separately and found that the effect on survival differed between the compartments. However, there was no consistency in subsite-specific effect of macrophage infiltration between the studies (Table 1). Meta-analysis of eligible studies showed a significant adverse effect of a high number of CD163+ M2 macrophages^42^ on overall survival (*P* < 0.0001) (Fig. 2a). Eliminating one study in turn did not alter this result, providing rather strong evidence for the robustness of this meta-analysis. On the contrary, cells detected by the pan-macrophage marker CD68 did not show significant association with overall survival in the meta-analysis (*P* = 0.22) (Fig. 2b).

Three studies assessed the prognostic value of activated NK cells using CD57 as a marker^29,43,44^ (Table 2), of which two were eligible for meta-analysis. In the meta-analysis, a high number of these cells were associated with a statistically significant survival benefit (*P* < 0.0001) (Fig. 2c). We found no evidence of between-study heterogeneity for neither CD57 nor for CD163. The single study that used the pan NK cell marker CD56 found no effect on survival^45^ (Table 2).

### T cells, B cells, DC and mast cells lack evidence of prognostic usefulness

Thirteen different studies assessed various subsets of T cells^23,28,29,34,40,44–51^ (Table 3). High numbers of tumour-infiltrating CD3+ T cells (pan T cell marker), CD4+ T cells (T helper (Th) cell marker) or CD8+ T cells (T cytotoxic (Tcyt) marker) were usually associated with somewhat longer survival, whereas high numbers of forkhead box P3 (FOXP3)+ T-regulatory cells showed significant association with decreased survival in two of the five studies addressing this cell type. Three CD8 studies were eligible for meta-analysis, which did not support a prognostic value for this cell type (*P* = 0.17) (Fig. 2d). There was evidence of between-study heterogeneity in the effect of CD8 (*I*^2^ = 76%, *P* = 0.02). We could not perform meta-analysis for the other T cell types due to lack of data.

B cells were analysed in five studies using four different markers^23,28,34,49,52^ (Table 3). B cells recognised by the pan B-cell markers CD19 and CD20 were mostly associated with survival benefits, although statistically significant in only two of the four studies. The single study assessing plasma cells neither reported the direction of effect on survival nor were the results statistically significant. B-regulatory cells were significantly associated with decreased survival in a single study but were not independent markers.

Seven different articles assessed the prognostic value of DC, using seven different markers^28,45,53–57^ (Table 2). High numbers of both immature CD209+ DC and plasmacytoid CD123+ DC were associated with decreased survival in one study, whereas high numbers of the other subsets of DC were mostly associated with improved survival. However, the results often lacked statistical significance. Studies that included tumour subsite-specific survival analyses of DC infiltration showed contradictory results (Table 2).

Two studies on mast cells^58,59^ passed our inclusion criteria. Both used mast cell tryptase as the cell marker, but the results were contradicting (Table 2). We could not perform meta-analysis for B cells, DC and mast cells because the studies lacked the required data.

### The reporting in the studies was often incomplete

**Fig. 3:**
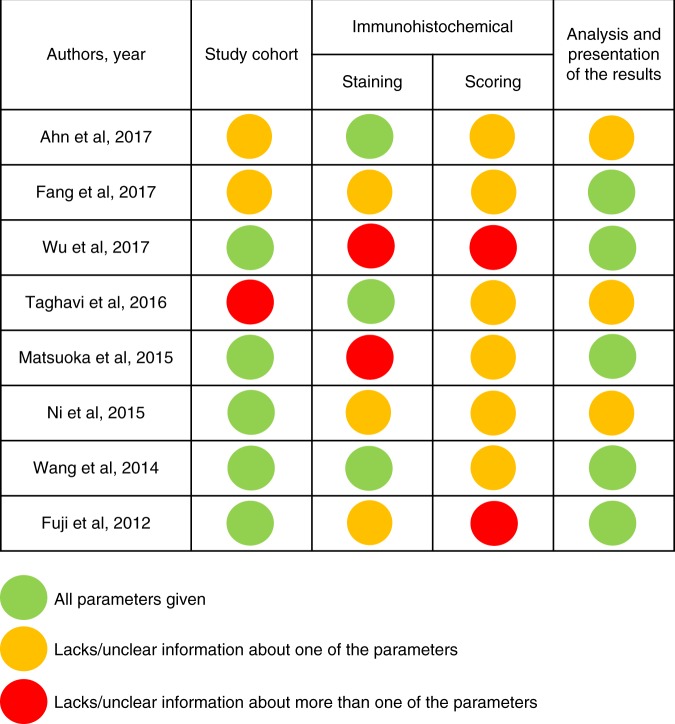
Assessment of the reporting quality of the studies included in the meta-analysis. The reporting of the following parameters was evaluated: Study cohort, (1) number of patients, (2) tumour size/stage, (3) chemotherapy/radiation of tissue prior to surgery; Immunohistochemical staining, (1) antibody clone/product number, (2) immunohistochemistry procedures, (3) positive and negative controls; scoring (1) number of observers, (2) clear scoring criteria, (3) inter/intra-observer variability; Analysis and presentation of the results, (1) survival endpoint analysed, (2) direction of effect on survival in Kaplan–Meier plot, (3) estimated effects with confidence intervals for the marker and, at least for the final model, all other variables in the model

We evaluated the reporting of the study cohort, the immunohistochemical staining and scoring procedures, as well as the analysis and presentation of the results for the studies included in the meta-analysis, based on a checklist adapted from the REMARK guidelines^14^ (Fig. 3). None of the studies completely adhered to our checklist, and there was a huge variation in the amount of information given and how it was presented. In general, the included studies scored higher in the categories “study cohort” and “analysis and presentation of the results” compared to “immunohistochemical staining and scoring”, where all studies lacked information about at least one parameter in the subcategory “scoring”. The information that was most often missing was the inter- and/or intra-observer variability for the different scorings, and the use of positive and negative controls for immunohistochemical staining. Other weaknesses included a lack of reporting of medical treatment prior to surgery and a lack of illustration of direction of survival in Kaplan–Meier plots. These weaknesses induce a risk of comparing groups with different baseline characteristics (selection bias), that received different care (performance bias), and where the results were interpreted in systematically different ways (detection bias). Contrary, we found no strong indication of reporting/publication bias, as many studies reported results that were not statistically significant (Tables 1–3).

## Discussion

OSCC are highly immunogenic tumours^5,60^ that are often characterised by abundant infiltration of immune cells. In this study, we have reviewed the current literature on the prognostic potential of tumour-infiltrating macrophages, DC, mast cells and lymphocytes in OSCC. We have also performed meta-analysis of studies that provided the necessary statistical data and have assessed the completeness of reporting of the study cohort, the immunohistochemical staining and scoring as well as the analysis and presentation of the results in these studies. CD163+ M2 macrophages and CD57+ NK cells were the only biomarkers that were statistically significant in pooled meta-analysis on overall survival and seem to have the best prognostic potential of the immune cell subsets addressed in this review.

A high count of tumour-infiltrating CD163+ M2 macrophages was significantly associated with decreased overall survival in pooled meta-analysis (Fig. 2a). Macrophages are phagocytic and antigen-presenting cells of the innate immune system, and are often among the most abundant immune cells in the tumour microenvironment.^61^ Macrophages can differentiate into several phenotypes depending on environmental cues. Classically activated or M1 macrophages can be induced by factors such as lipopolysaccharides, interferon gamma (INFɣ) and tumour necrosis factor (TNF). These cells can produce pro-inflammatory cytokines, stimulate a Th1 immune response and are thought to be tumour-suppressive. Alternatively activated or M2 macrophages can be induced by interleukin (IL) 4, -10 and -13, as well as by corticosteroids and prostaglandin E. These cells are involved in tissue repair, angiogenesis and immunosuppression, and can promote tumour growth.^61,62^ However, recent evidence suggests that there is a continuum of phenotypes between the “pure” M1 and M2 macrophages, and that the cells may be redirected from one phenotype towards the other.^63^ Tumour-associated macrophages are often differentiated towards the tumour-supporting M2 phenotype, which is also reflected by our results where high counts of CD68+ and CD163+ cells were mostly associated with decreased survival. However, only CD163 was statistically significant in pooled meta-analysis, indicating that CD163 is a more reliable prognostic marker in OSCC than CD68. This might indicate that there is a mixture of macrophage subtypes in the tumour, where some of those recognised by the pan-macrophage marker CD68 may have tumour-suppressive activities. Furthermore, cell types other than macrophages, such as subtypes of lymphocytes, may also express CD68.^64^ This may further weaken the prognostic strength of this marker.

In contrast to CD163, a high count of tumour-infiltrating CD57+ NK cells was significantly associated with improved overall survival in pooled meta-analysis (Fig. 2c). NK cells are important effector cells of the innate immune system. They share many common features with Tcyt cells, but in in contrast to Tcyt cells, they can kill cells lacking the self-recognition marker major histocompatibility complex 1 without further activation.^65^ NK cells also produce cytokines such as INFɣ that activate other immune cells, including M1 macrophages.^11,66^ This supports the NK cells’ role as potent tumour suppressors and putative prognostic markers. As for most other immune cells, there are several subtypes of NK cells, where the two main populations are the CD56^bright^ and the CD56^dim^. CD56^dim^ NK cells have a higher expression of proteins such as perforin and granzymes than CD56^bright^ and are therefore more cytotoxic. In contrast, the CD56^bright^ NK cells produce higher amounts of INFɣ, and have more potent immune regulatory functions.^67^ Based on the available data, CD57+ was the only significant biomarker for NK cells. Only CD56^dim^ NK cells express CD57,^67,68^ thus this marker may more precisely detect NK cells with high cytotoxic activity. The only study that assessed the prognostic value of NK cells recognised by the pan NK cell marker CD56 found no statistically significant survival effect of these cells. Notably, all studies on CD163 and CD57 included in the meta-analysis were performed on an East-Asian patient cohort (Tables 1 and 2), and ethnical and cultural differences along with varying access to health care and diverse treatment regimens call for care when extrapolating results based on patients from one part of the world to others. Thus, well-controlled studies are needed to confirm the robustness and relevance of CD163+ and CD57+ cells as prognostic markers in OSCC patient cohorts outside of Asia.

The other cell types addressed in this review lack evidence of prognostic usefulness. Most of these immune cells can be divided into a number of different subclasses with distinct functional properties. This was most notable for DC analysed with seven different markers (Table 2) in the seven articles on DC eligible for this review. DC are potent antigen-presenting cells and important linkers between the innate and the adaptive immune system. Plasmacytoid DC are derived from lymphoid progenitor cells and have an important role in the fight against viral infections. CD123 is often used as a marker for plasmacytoid DC, but the protein is also expressed by pre-DC that can differentiate into other DC subtypes.^69^ In addition, there are two main classes of conventional (c) DC derived from myeloid progenitor cells, cDC1 and cDC2. cDC1 cells express high levels of CD141 and low levels of CD11b and CD11c.^70^ They are potent activators of CD8+ Tcyt cells and promote a Th1 response. Compared to cDC1 cells, cDC2 cells are less abundant, and express high levels of markers such as CD1c, CD11b and CD11c. Several subsets of cDC2 cells exist that can elicit a wide range of responses, including both Th1 and Th2 activation.^69^ In addition to DC originating from lymphoid and myeloid progenitor cells, DC can also derive from monocytes under inflammatory conditions.^71^ Monocyte-derived DC express high levels of CD11c and CD1a. CD1a is however also expressed by Langerhans cells, specialised DC found in the basal layer of stratified squamous epithelium. Functionally, Langerhans cells seem to have much in common with cDC1 cells.^72^ Different activation/maturation statuses can further influence the functional properties of the various DC subtypes.^69^ To determine DC subtype and activation status, requires detection of several markers simultaneously by double-, triple or even quadruple immunohistochemical staining. This is technically demanding, and none of the DC studies reviewed used more than one marker at a time. Furthermore, cell types other than DC can be positive for the markers used, such as S100 that is expressed by a broad range of cell types. Thus, based on our results from the reviewed literature, it is impossible to draw reliable conclusions about the prognostic potential of the various DC subtypes. The diversity of DC makes precise immunohistochemical identification of DC subtypes complicated and questions the feasibility of using DC immunohistochemical detection for prognostic purposes.

Mast cells are of hematopoietic origin, and are loaded with granules containing bioactive molecules such as growth factors, interleukins, chemokines and proteases, of which mast cell tryptase is often used as a mast cell marker.^73^ Upon activation through stimuli such as immune receptors, pathogens or endogenous compounds, mast cells can release these molecules and initiate a wide range of immune responses.^74^ Mast cells are linked to immunoglobulin E-mediated immune responses, but are increasingly recognised to serve multiple tissue functions including regulation of blood flow, wound healing as well as innate and adaptive immune responses.^75^ Through crosstalk with B cells, T cells and DC, mast cells may have important regulatory functions.^74^ In human solid tumours, mast cells are believed to have dual effects. Secretion of IL-8 and vascular endothelial growth factor can promote angiogenesis and tumour growth, whereas release of cytotoxic cytokines and TNFα may have tumour-suppressive effects.^76^ Neither of the two studies addressing mast cells in our review found them to be independent predictors of patient survival. However, as for many other immune cells, it could be that the full spectrum of mast cell diversity is yet to be elucidated. Data on the function and prognostic value of mast cells in human tumours are sparse, and more studies are warranted.

T cells and B cells are the main cells of the adaptive immune response. T cells, recognised by the pan T cell marker CD3, are broadly divided into CD4+ T helper (Th) cells and CD8+ Tcyt cells.^77^ The CD4+ Th cells produce an array of cytokines that modulate neighbouring immune cell function, and release chemokines that attract inflammatory cells. Dependent on the stimuli, CD4+ Th cells can differentiate into a number of subsets including Th1, Th2, Th9, Th17 and Treg cells, which each release specific cytokines and chemokines and thereby have distinct functions. The Th1 response is triggered by cytokines such as INF α/β and IL-12, and is characterised by release of large amounts of INFɣ and TNFα.^78^ This in turn may stimulate generation of M1 macrophages and suppress tumour growth.^61^ Th2 cells are differentiated in response to IL-4, whereupon the Th2 cells produce vast amounts of IL-4, -5 and 13. These cytokines can stimulate basophils, mast cells and eosinophils, as well as promote differentiation of tumour-promoting M2 macrophages.^79^ TGFβ can promote differentiation of Treg cells, often recognised by expression of the transcription factor FOXP3. Treg cells also produce TGFβ as well as IL-12, which inhibit T cell proliferation and cytokine release, thereby dampening the immune response.^80^ This could represent a mechanism of conveying immune surveillance of a tumour, yet, the studies included in this review show contradicting result on the prognostic value of FOXP3+ cells. As for the DC, a panel of antibodies would be needed to accurately identify the presence and localisation of the numerous CD4+ T cell subtypes. Merely staining with CD3 or CD4 recognises cells with a broad range of functions and may therefore have limited prognostic usefulness.

For CD8+ T cells, fewer functional subtypes have been recognised than for CD4+ T cells. Similar to Th1 cells, CD8+ Tcyt cells are stimulated by IL-12 and INFα/β. Upon stimulation, they produce INFɣ and TNFα in addition to granzymes and perforin, which can lyse and kill neighbouring cells.^81^ Thus, Tcyt cells may efficiently kill tumour cells, and have important tumour suppressor functions. However, signalling through receptors such as CTLA-4 and PD-1 on Tcyt cells may inhibit their cytotoxic activity.^82^ This could explain why we did not find any statistically significant association between CD8+ cells and survival in the meta-analysis.

B cells are another major player in the adaptive immune response, more specifically in the humoral response. B cells are often recognised by the pan markers CD19 or CD20.^83^ Upon binding of an antigen compatible with their specific receptor, and supported by cytokines released from Th cells, B cells can differentiate to plasma cells and produce antibodies specific to the antigen that triggered their differentiation.^84^ However, B cells also act as antigen-presenting cells, promote differentiation of Th1 cells and Tcyt cells, and directly kill cancer cells through release of Granzyme B.^85^ These functions support a tumour-suppressive role for B cells. However, as for T cells, immune-suppressive subtypes of B cells have been detected, termed B-regulatory (Breg) cells. Similar to Treg cells, these cells produce TGFβ and IL-10, promoting the same down-stream effects as described for Treg cells.^83^ This heterogeneity in B-cell function may explain the lack of consensus in results on cells using pan B-cell markers. The single study exploring Breg cells showed a negative but not independent effect on survival. B-cell maturation and activation normally happens in secondary lymphoid organs but may also take place in tertiary lymphoid structures that are aggregates of lymphocytes organised as lymphoid follicles at sites of inflammation. Such structures have been found in several types of cancer including OSCC,^86^ where they may act as sites of T cell and B-cell activation and antibody production in the tumour microenvironment. This suggests that the function of immune cells may depend on how these cells are organised in the tumour microenvironment.^73–76^

The anatomical location of the tumour within the oral cavity may also influence the prognostic value of immune cells. Most of the studies in this review included patients with cancers at various intraoral locations, but hardly any of them reported subsite-specific prognostic data. More often, various tumour compartments were analysed for immune cell infiltration, e.g. the invasive front, the tumour periphery or the tumour nests (Tables 1–3). However, the definition of these compartments was often unclear, making it difficult to compare the results. Furthermore, some studies used resection samples whereas others used tissue microarrays or biopsy samples, where the latter may be less representative of the whole tumour.

The reliability of our results is limited by reporting deficiencies in the included studies, which we assessed based on a checklist adapted from the REMARK guidelines. All studies included in our meta-analysis were published at least 7 years after the REMARK guidelines were first introduced in 2005, and most of them were conducted after an elaboration of the REMARK guidelines was published in 2012.^87^ None of the studies included in this review provided all of the recommended information (Fig. 3), and the reporting of immunohistochemical staining and scoring protocols was insufficient in most of the studies. This, along with results from a recent study^88^ indicate that the use of the REMARK guidelines is yet to be implemented in research practice. In 2018, an abridged explanation and elaboration of the REMARK guidelines was published to encourage dissemination of the REMARK checklist,^89^ and it will be interesting to see if adherence of the REMARK guidelines improves in the scientific community. Proper reporting of the study cohort and—design, assay methods, statistical analyses and results allows transparency and reliability of prognostic marker studies.

Several of the reviewed papers merely stated whether a marker was statistically significant or not based on a *P*-value and significance level but did not report the *P*-value itself or the direction of the effect on survival. However, small differences in study design such as the inclusion or exclusion of patients can shift the *P*-value above or below the common 0.05 level of significance.^90^ Especially in small studies and when controlling for multiple parameters, the prognostic value of a marker is likely to be underestimated. Thus, evaluating the effect size and direction on survival may be a more suitable approach to estimate the prognostic value of a marker.^90^ When extracting data for this systematic review, we tried to interpret whether infiltration of the various cell types was associated with longer or shorter survival, independent of the statistical significance of the association, as indicated in Tables 1–3. Importantly, the effect on survival in many of the studies was minor, and conclusions derived from these studies may have poor reliability. Only a small number of studies reported the necessary statistical data to be eligible for meta-analysis, which limits the reliability of our conclusions. More rigorous reporting of statistical data will help to increase the quality of individual studies and opens the possibility to summarise the existing data in meta-analyses.

## Conclusion

Important clinical information or reporting of immunohistochemical staining and scoring protocols as well as statistical data were often missing in the papers reviewed in this study. Thus, there is a need for journal editors and reviewers to focus more on the quality of data reporting in prognostic marker studies to increase their impact and usefulness. With these methodological limitations in mind, we conclude that M2 macrophages identified by CD163 and mature NK cells identified by CD57 seem to be the most promising prognostic factors of the immune cells analysed in this review. However, the prognostic value of CD163+ M2 macrophages and CD57+ NK cells should be confirmed in a large cohort of OSCC patients with transparent and comprehensive reporting of study design and conduct. All other evaluated cells show conflicting or statistically insignificant results, and their potential as prognostic markers is far from being established.

## Supplementary information

Supplementary Figure 1

## Data Availability

All data reported in this manuscript are found in the literature as cited in the text.
