# Supplementary material for: Tissue-infiltrating immune cells as prognostic markers in oral squamous cell carcinoma: a systematic review and meta-analysis
Source: Br J Cancer. 2019 Feb 27;120(7):714–27. doi: 10.1038/s41416-019-0409-6 (PMC6461751; doi:10.1038/s41416-019-0409-6)
Supplement: Supplementary file 1 — Supplementary Figure 1 [file 41416_2019_409_MOESM1_ESM.pptx]

## Slide 1
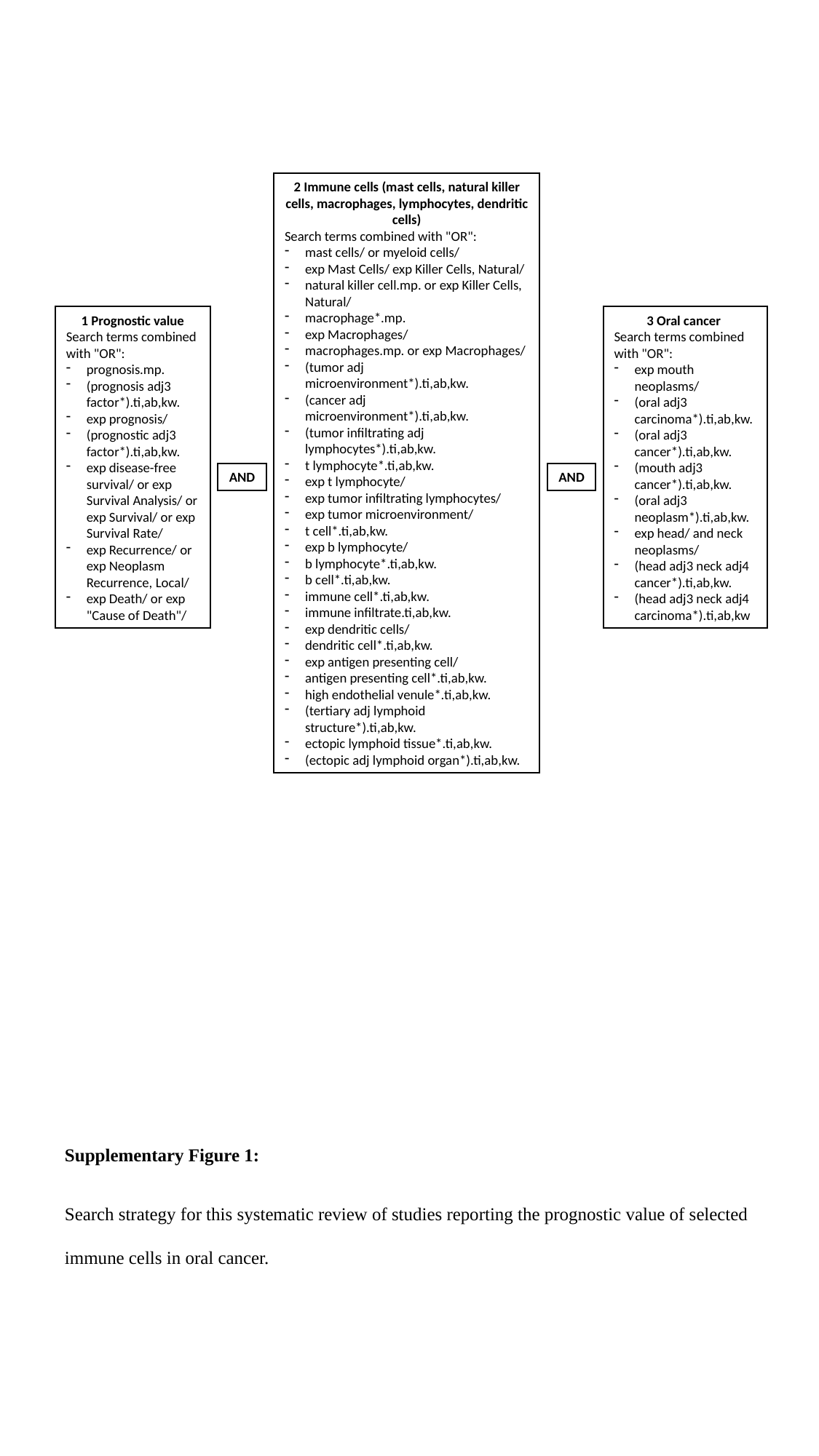

2 Immune cells (mast cells, natural killer cells, macrophages, lymphocytes, dendritic cells)
Search terms combined with "OR":
mast cells/ or myeloid cells/
exp Mast Cells/ exp Killer Cells, Natural/
natural killer cell.mp. or exp Killer Cells, Natural/
macrophage*.mp.
exp Macrophages/
macrophages.mp. or exp Macrophages/
(tumor adj microenvironment*).ti,ab,kw.
(cancer adj microenvironment*).ti,ab,kw.
(tumor infiltrating adj lymphocytes*).ti,ab,kw.
t lymphocyte*.ti,ab,kw.
exp t lymphocyte/
exp tumor infiltrating lymphocytes/
exp tumor microenvironment/
t cell*.ti,ab,kw.
exp b lymphocyte/
b lymphocyte*.ti,ab,kw.
b cell*.ti,ab,kw.
immune cell*.ti,ab,kw.
immune infiltrate.ti,ab,kw.
exp dendritic cells/
dendritic cell*.ti,ab,kw.
exp antigen presenting cell/
antigen presenting cell*.ti,ab,kw.
high endothelial venule*.ti,ab,kw.
(tertiary adj lymphoid structure*).ti,ab,kw.
ectopic lymphoid tissue*.ti,ab,kw.
(ectopic adj lymphoid organ*).ti,ab,kw.
1 Prognostic value
Search terms combined with "OR":
prognosis.mp.
(prognosis adj3 factor*).ti,ab,kw.
exp prognosis/
(prognostic adj3 factor*).ti,ab,kw.
exp disease-free survival/ or exp Survival Analysis/ or exp Survival/ or exp Survival Rate/
exp Recurrence/ or exp Neoplasm Recurrence, Local/
exp Death/ or exp "Cause of Death"/
3 Oral cancer
Search terms combined with "OR":
exp mouth neoplasms/
(oral adj3 carcinoma*).ti,ab,kw.
(oral adj3 cancer*).ti,ab,kw.
(mouth adj3 cancer*).ti,ab,kw.
(oral adj3 neoplasm*).ti,ab,kw.
exp head/ and neck neoplasms/
(head adj3 neck adj4 cancer*).ti,ab,kw.
(head adj3 neck adj4 carcinoma*).ti,ab,kw
AND
AND
Supplementary Figure 1:
Search strategy for this systematic review of studies reporting the prognostic value of selected immune cells in oral cancer.
